# Differential roles of NF-Y transcription factor in ER chaperone expression and neuronal maintenance in the CNS

**DOI:** 10.1038/srep34575

**Published:** 2016-09-30

**Authors:** Tomoyuki Yamanaka, Asako Tosaki, Haruko Miyazaki, Masaru Kurosawa, Masato Koike, Yasuo Uchiyama, Sankar N. Maity, Hidemi Misawa, Ryosuke Takahashi, Tomomi Shimogori, Nobutaka Hattori, Nobuyuki Nukina

**Affiliations:** 1 Laboratory of Structural Neuropathology, Doshisha University Graduate School of Brain Science, Kyoto 610-0394, Japan; 2 Laboratory for Structural Neuropathology, RIKEN Brain Science Institute, Saitama 351-0198, Japan; 3 Department of Neuroscience for Neurodegenerative Disorders, Juntendo University Graduate School of Medicine, Tokyo 113-8421, Japan; 4 Laboratory for Molecular Mechanisms of Thalamus Development, RIKEN Brain Science Institute, Saitama 351-0198, Japan; 5 Department of Cell Biology and Neuroscience, Juntendo University Graduate School of Medicine, Tokyo 113-8421, Japan; 6 Departments of Cellular and Molecular Neuropathology, Juntendo University Graduate School of Medicine, Tokyo 113-8421, Japan; 7 Department of Genitourinary Medical Oncology, The University of Texas MD Anderson Cancer Center, Houston, Texas 77030, USA; 8 Division of Pharmacology, Faculty of Pharmacy, Keio University, Tokyo 105–8512, Japan; 9 Department of Neurology, Graduate School of Medicine, Kyoto University, Kyoto 606-8507, Japan; 10 Department of Neurology, Juntendo University Graduate School of Medicine, Tokyo 113-8421, Japan

## Abstract

The mammalian central nervous system (CNS) contains various types of neurons with different neuronal functions. In contrast to established roles of cell type-specific transcription factors on neuronal specification and maintenance, whether ubiquitous transcription factors have conserved or differential neuronal function remains uncertain. Here, we revealed that inactivation of a ubiquitous factor NF-Y in different sets of neurons resulted in cell type-specific neuropathologies and gene downregulation in mouse CNS. In striatal and cerebellar neurons, NF-Y inactivation led to ubiquitin/p62 pathologies with downregulation of an endoplasmic reticulum (ER) chaperone Grp94, as we previously observed by NF-Y deletion in cortical neurons. In contrast, NF-Y inactivation in motor neurons induced neuronal loss without obvious protein deposition. Detailed analysis clarified downregulation of another ER chaperone Grp78 in addition to Grp94 in motor neurons, and knockdown of both ER chaperones in motor neurons recapitulated the pathology observed after NF-Y inactivation. Finally, additional downregulation of Grp78 in striatal neurons suppressed ubiquitin accumulation induced by NF-Y inactivation, implying that selective ER chaperone downregulation mediates different neuropathologies. Our data suggest distinct roles of NF-Y in protein homeostasis and neuronal maintenance in the CNS by differential regulation of ER chaperone expression.

The mammalian central nervous system (CNS) contains various types of neurons with differential distributions. They differ in morphology, size, neuronal connections and cellular contents, enabling them to exert specific neuronal functions. Neuronal type-specific transcription factors, called master factors, have been shown to play critical roles in generation and maintenance of the neurons by transcribing specific sets of genes^1^. On the other hands, recent observations identified significance of ubiquitous transcription factors in neuronal differentiation and maintenance^2^,^3^,^4^,^5^,^6^,^7^,^8^,^9^,^10^. However, it is still uncertain whether the ubiquitous factors have conserved or differential roles in multiple types of CNS neurons.

Nuclear factor-Y (NF-Y), also known as the CCAAT binding factor, is a ubiquitous transcription factor composed of three subunits: NF-YA, NF-YB and NF-YC^11^,^12^. NF-Y has been shown to be a key regulator of cell-cycle progression in various types of proliferating cells including fibroblasts, embryonic stem (ES) cells, hematopoietic stem cells, myoblasts and tumor cells^13^,^14^,^15^,^16^,^17^,^18^. In contrast, the role of NF-Y in non-proliferative cells remained to be clarified because the activity is often lost after differentiation^15^,^16^. However, we previously found that NF-Y is still active in mature neurons and furthermore is suppressed during neurodegeneration in brain of Huntington’s disease model mouse through sequestration of NF-YA by expanded polyglutamine protein^19^. The NF-YA sequestration has also been reported in other neurodegenerative diseases caused by polyglutamine expansion, such as spinal and bulbar muscular atrophy and spinocerebellar ataxia 17^20^,^21^. Because different brain regions are selectively affected in these polyglutamine diseases^22^,^23^, these observations suggest a potential role of NF-Y in maintenance/degeneration of multiple types of CNS neurons.

We recently reported that conditional deletion of NF-YA by a camk2a-cre transgene induces degeneration of cerebral pyramidal neurons in mouse brain^2^. Interestingly, the degeneration accompanies accumulation of insoluble membrane proteins on disorganized endoplasmic reticulum (ER) together with ubiquitin (Ub) and p62, as well as downregulation of several ER-related genes such as an ER chaperone Grp94. These data clearly indicate the neuron-specific function of NF-Y for cell maintenance possibly by regulating protein homeostasis in ER. In addition to the cell maintenance, an *in vitro* study using ES cells suggested a role of NF-Y in neural specification during differentiation^6^. Although these observations indicate the significance of NF-Y in neuronal maintenance and differentiation, it is unclear whether its neuronal functions are preserved in multiple types of neurons in different CNS regions.

In this study, we inactivated NF-Y in different sets of CNS neurons including striatal medium spiny neurons (MSNs), cerebellar Purkinje cells and spinal/brainstem motor neurons by gene knockdown and knockout techniques. Although all three neuronal types were degenerated after NF-Y inactivation, the pathological phenotypes were different. Whereas the MSNs and Purkinje cells developed Ub/p62 pathologies similar to those observed in the NF-YA knockout pyramidal neurons, the motor neurons did not. Further analysis revealed that, in contrast to the former two neurons showing only Grp94 downregulation, the motor neurons lost expression of another major ER chaperone, Grp78. We observed that knockdown of both Grp78 and Grp94 in motor neurons was sufficient to recapitulate the pathology observed by NF-YA knockout. Finally, additional downregulation of Grp78 in striatal MSNs suppressed Ub accumulation after NF-Y inactivation. These data indicate that NF-Y is differentially involved in protein homeostasis and neuronal maintenance in the CNS by expressing different sets of ER chaperones. We thus propose that, not only the neuronal type-specific transcription factors^1^, the ubiquitous factor NF-Y also has neuronal type-specific roles in the CNS.

## Results

### NF-YA deletion in motor neurons induces progressive neurodegeneration

To explore the role of NF-Y in multiple CNS neurons, we first focused on motor neurons because there is a well-established transgenic line, VAChT-cre (VA-cre), which expresses cre recombinase specifically in motor neurons of spinal cord and brainstem after birth^24^. By crossing the VA-cre mice with NF-YA flox mice, we generated motor-neuron-specific NF-YA deletion (NF-YA flox/flox; VA-cre) mice (hereafter referred to as NF-YA v-cko mice). The apparent phenotypes of the v-cko mice were abnormal posture (Supplementary Fig. 1a) and progressive weight loss (Supplementary Fig. 1b). In addition, the v-cko mice showed tremor-like movement that became more prominent with aging (Supplementary Fig. 1c,d).

**Figure 1:**
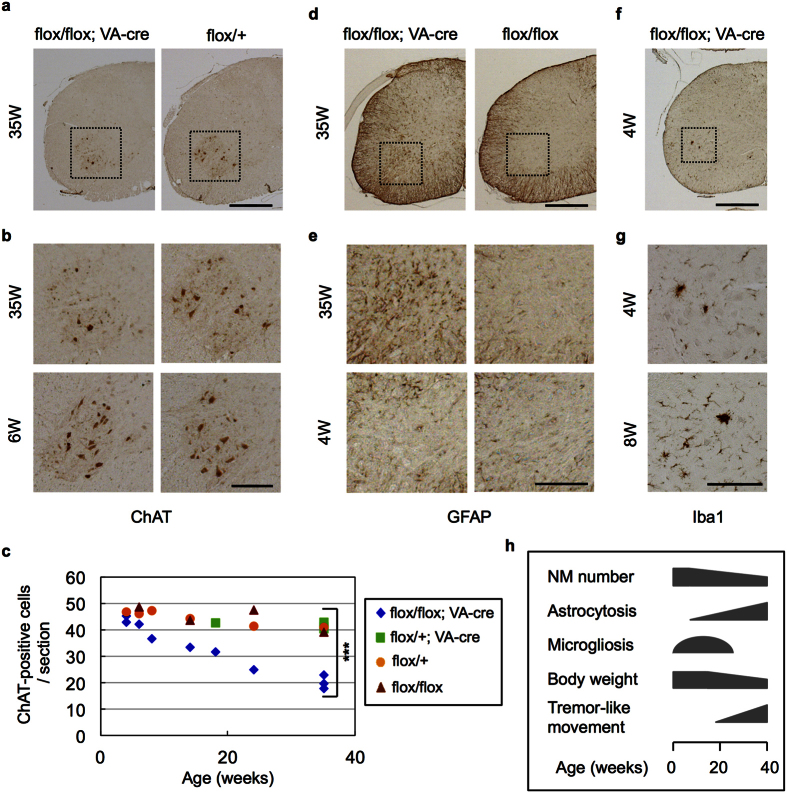
Progressive motor neuron loss and glyosis induction in anterior horn of NF-YA v-cko mice. Immunohistochemistry of cervical spinal cord of NF-YA v-cko mice (flox/flox; VA-cre) and control mice (flox/+ or flox/flox or flox/+; VA-cre) at indicated weeks of age. (**a**) Anti-ChAT staining. (**b**) Enlarged images of boxed regions in (**a**). Note the reduction of ChAT-positive motor neurons in anterior horn of NF-YA v-cko mice at 35 weeks but not at 6 weeks of age. (**c**) Quantification of ChAT-positive cells in cervical cord sections of mice with indicated genotypes. Mean of cell numbers in eight sections are shown. A statistical analysis was performed on data from v-cko (flox/flox; VA-cre) and control mice (flox/+; VA-cre) at 35 weeks of age (***P < 0.001, t-test). Note the progressive reduction of ChAT-positive neurons in NF-YA v-cko mice. (**d**) Anti-GFAP staining. (**e**) Enlarged images of anterior horns indicated in (**d**). (**f**) Anti-Iba1 staining. (**g**) Enlarged images of anterior horns indicated in (**f**). Note the induction of astrocytosis and microgliosis in anterior horns of NF-YA v-cko mice. (**h**) Summary of the age-dependent phenotypes of NF-YA v-cko mice. Scale bars are 500 μm (**a**,**d**,**f**), 200 μm (**b**,**e**,**g**).

Histological analysis identified reduction of choline acetyltransferase (ChAT)-positive motor neurons in cervical anterior horns of v-cko mice at 35 weeks of age but not those at 6 weeks of age (Fig. 1a,b). Quantitative analysis of mice of different ages revealed progressive reduction of the motor neurons (Fig. 1c), finally reaching half of the control number, probably due to the restricted cre expression to around 50% of motor neurons^24^. We further observed astrocytosis in anterior horns of v-cko mice, which became more severe with aging (Fig. 1d,e). Microgliosis was also observed in the anterior horns of the v-cko mice at earlier stages of degeneration (4–8 weeks of age; Fig. 1f,g) but it was hardly detected at later, fully degenerated stages (24–35 weeks of age; data not shown). Astrocytosis and microgliosis were also observed in facial motor nuclei of brainstem in NF-YA v-cko mice (Supplementary Fig. 2). Taken together, these data indicate that NF-Y is indispensable for motor neuron maintenance and its inactivation through NF-YA deletion induces progressive neurodegeneration accompanying gliosis (Fig. 1h).

### No accumulation of Ub or p62 in motor neurons by NF-YA deletion

**Figure 2:**
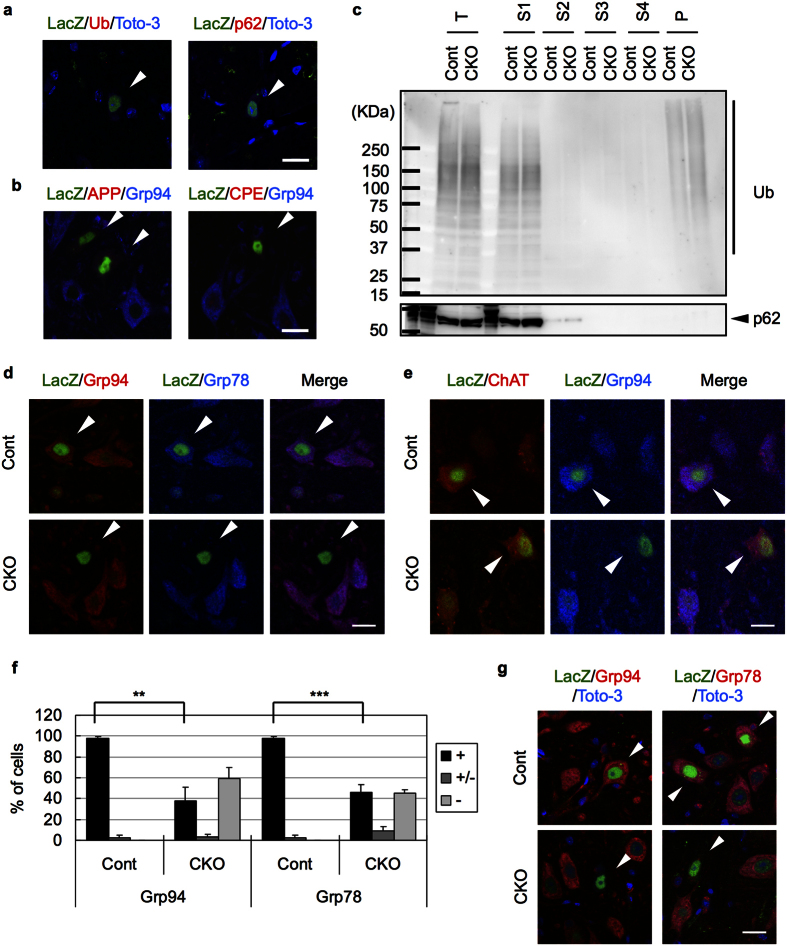
No accumulation of Ub/p62 and loss of ER chaperones in NF-YA v-cko motor neurons. (**a**,**b**) Cervical cord sections of 6-week-old NF-YA v-cko mice (flox/flox; VA-cre; RNZ) were stained with LacZ together with indicated antibodies. Nuclei were stained with Toto-3 for (**a**). LacZ-positive cells are indicated by arrowheads. Note no accumulation of Ub, p62, APP or CPE in NF-YA v-cko motor neurons. (**c**) Isolated spinal cords from 14-week-old NF-YA v-cko mice (flox/flox; VA-cre) and control mice (flox/+) were homogenized (T), and then sequentially solubilized with buffer containing 1% TritonX-100 (S1), DNaseI/RNaseA (S2), 1% Sarkosyl (S3) or 4% Sarkosyl (S4), or with formic acid (P), and then analyzed by western blotting. Note no accumulation of insoluble Ub or p62 in NF-YA v-cko spinal cords. (**d**,**e**) Cervical cord sections of 6-week-old NF-YA v-cko (flox/flox; VA-cre; RNZ) or control (flox/+; VA-cre, RNZ) mice were stained with LacZ together with indicated antibodies. LacZ-positive cells are indicated by arrowheads. (**d**) Loss of both Grp94 and Grp78 in NF-YA v-cko neurons. (**e**) Remaining ChAT expression in the NF-YA v-cko neurons without Grp94 expression. (**f**) The LacZ-positive cells were categorized into three groups based on the staining of Grp94 or Grp78; no signal (−), background level (+/−) or similar to the control level (+). Ratios to total counted cells are shown. Values are means + s.d. of four mice data (**P < 0.01, ***P < 0.001, t-test). (**g**) Facial nuclei of the mice used in a were stained with LacZ together with Grp94 or Grp78. Nuclei were stained with Toto-3. Loss of these ER chaperones in NF-YA v-cko facial motor neurons. Scale bars are 20 μm.

We have previously shown that NF-YA deletion in cerebral pyramidal neurons induces accumulation of insoluble Ub, p62 and several membrane proteins such as amyloid precursor protein (APP) and carboxypeptidase E (CPE) during neurodegeneration^2^. To test whether motor neuron degeneration also accompanied the abnormal protein accumulation, we analyzed v-cko mice harboring an RNZ reporter that expresses nuclear LacZ by cre-mediated recombination^25^. Staining of the spinal cord sections revealed no accumulation of Ub or p62 in LacZ-positive neurons of v-cko mice (Fig. 2a). Neither APP nor CPE accumulated in the LacZ-positive neurons, whereas they lost Grp94, a downstream target of NF-Y (Fig. 2b). We then sequentially fractionated v-cko mice spinal cords with different detergent buffers and found no accumulation of Ub or p62 in any fractions (Fig. 2c). Taken together, these data indicate that Ub, p62 or membrane proteins are not accumulated or insolubilized in motor neurons of NF-YA v-cko mice.

### Downregulation of multiple ER chaperones in NF-YA v-cko motor neurons

NF-Y binds to the promoters of Grp78 and Grp94^2^,^26^,^27^. Whereas NF-Y inactivation induces selective downregulation of Grp94 but not Grp78 in cerebral pyramidal neurons^2^, Grp78 downregulation after NF-Y suppression has been reported in other types of cells^27^,^28^, suggesting context-dependent regulation of Grp78 expression. Notably, staining of spinal cords of NF-YA v-cko mice showed coordinated loss of both Grp78 and Grp94 in LacZ-positive motor neurons (Fig. 2d). Quantification revealed that around 60% of LacZ-positive neurons showed severe reduction of both ER chaperones (Fig. 2f). In contrast, the Grp94-negative neurons were still positive for ChAT (Fig. 2e), suggesting that loss of ER chaperones is not simply due to loss of neuronal property. The downregulation of Grp78/94 was also observed in motor neurons of facial nuclei (Fig. 2g). Taken together, these data indicate that in contrast to the pyramidal neurons, the motor neurons lost expression of both ER chaperones upon inactivation of NF-Y.

We also found reduced staining of anti-KDEL, which recognizes proteins with a C-terminal KDEL motif such as Grp78 and Grp94 (Supplementary Fig. 3a). Protein disulfide isomerase (PDI), which functions as an enzyme and chaperone in ER lumen, was also absent in LacZ-positive v-cko neurons (Supplementary Fig. 3b). In contrast, the ER membrane protein Sec61β remained (Supplementary Fig. 3c), suggesting that the observed loss of luminal proteins was not due to the disappearance of ER itself. Considering the NF-Y binding to promoters of several ER chaperones including PDI^26^, NF-Y inactivation may induce global downregulation of ER chaperones in motor neurons. As for cytoplasmic chaperones, only Hsp70, a well-known NF-Y target^19^, was downregulated (Supplementary Fig. 3d). Other chaperones located in cytoplasm (Hsp90, Hsp40s (Hdj1/2)) and in mitochondria (Hsp60) remained to be expressed in LacZ-positive motor neurons without Grp94 expression (Supplementary Fig. 3e–h). Taken together, these data indicate that expression of ER chaperones is preferentially affected by NF-Y inactivation in motor neurons.

### Abnormal nuclear morphology in motor neurons by NF-YA deletion and Grp78/94 downregulation

**Figure 3:**
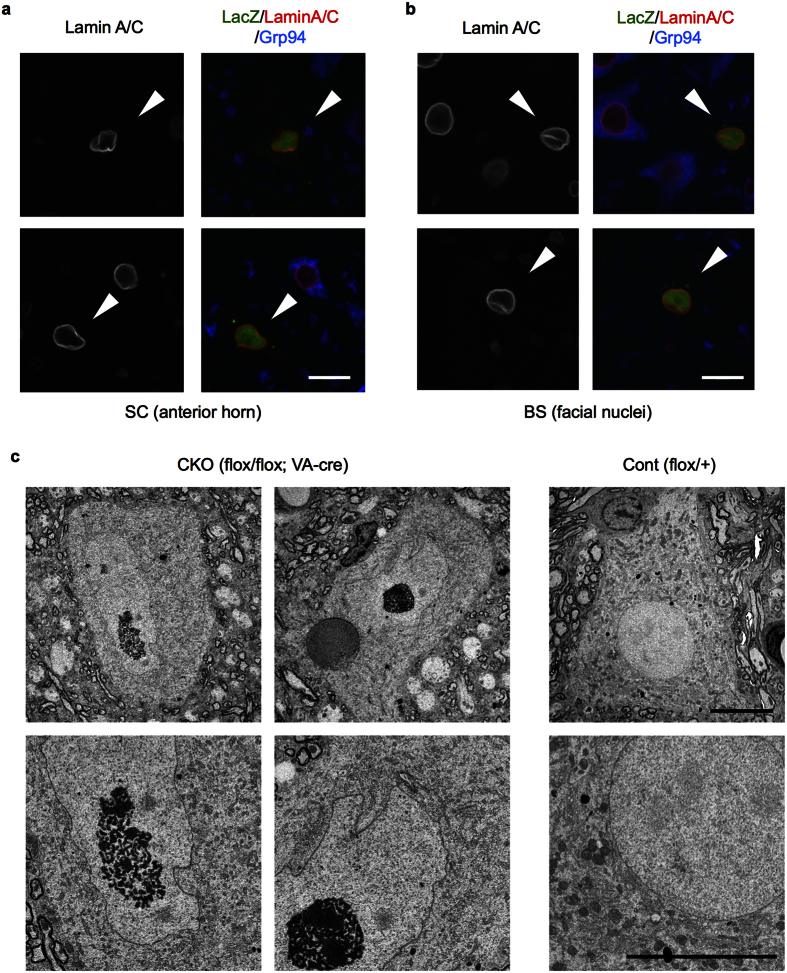
Abnormal nuclear morphology in NF-YA v-cko motor neurons. (**a**,**b**) Sections of cervical cord (**a**) or brain stem (**b**) of 6-week-old NF-YA cko mice (flox/flox; VA-cre; RNZ) were stained with LacZ, lamin A/C and Grp94, and analyzed by confocal microscopy. Note the abnormal nuclear morphology of LacZ-positive cells without Grp94 staining. (**c**) Electron micrographs of spinal cord motor neurons in 5-week-old NF-YA v-cko (flox/flox, VA-cre) or Cont (flox/+) mice. Lower panels are enlarged images of upper ones. Note the abnormal nuclear morphology of motor neurons of NF-YA v-cko mice, compared to normal and rounded nuclei in those of control mice. Scale bars are 20 μm (**a**,**b**) and 10 μm (**c**).

Although motor neurons did not show Ub/p62 pathology, staining of the nuclear lamin suggested that in NF-YA v-cko mice spinal and facial motor neurons positive for LacZ contained nuclei with abnormal morphologies when compared with LacZ-negative control cells (Fig. 3a,b). EM analysis of the spinal motor neurons revealed that in contrast to the control motor neurons with round-shaped nuclei, the nuclei of v-cko motor neurons were relatively elongated with multiple indentations (Fig. 3c). EM analysis also identified other abnormalities including cytosolic particles containing densely stained material, resembling lipid droplets, and aggregated ER membranes (Fig. 3c, data not shown).

**Figure 4:**
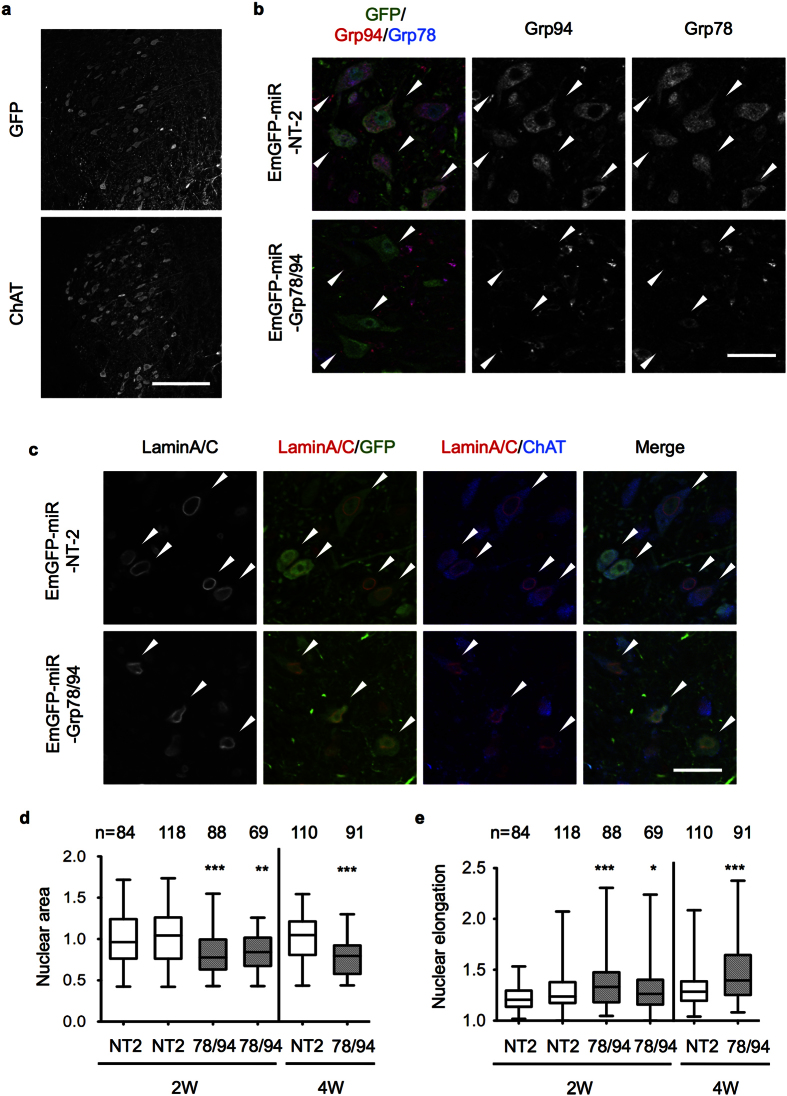
Knockdown of both Grp78 and Grp94 induces nuclear morphological alteration in trigeminal motor neurons. Mixed AAVs for EmGFP-miR-Grp78 and -Grp94 or AAV for EmGFP-miR-NT-2 (non targeting control) was injected into trigeminal nuclei of brain stem in wild type B6 mice. Mice were fixed at 2 weeks after injection and coronal sections were stained with indicated antibodies. (**a**) Co-staining of the sections of AAV-EmGFP-miR-NT-2-injected brain with GFP and ChAT. A portion of the ChAT-positive trigeminal motor neurons was expressing EmGFP. (**b**) Loss of both Grp78 and Grp94 in EmGFP-positive, miR-Grp78/Grp94-expressing trigeminal motor neurons. (**c**) Altered nuclear morphology by expression of miR-Grp78/Grp94 but not by that of NT-2. (**d**,**e**) Quantification of nuclear area (**d**) and elongation (ratio of long to short axis) (**e**) in the EmGFP-positive motor neurons of trigeminal nuclei at two or four weeks after AAV injection (n means number of analyzed cells). As for nuclear area, ratios to control (NT-2-expressing cells) are shown. The data are plotted in a box (25–75th percentile) and whisker chart. For statistical analysis we used ANOVA and t-test for data from two and four weeks, respectively (*P < 0.05, **P < 0.01, ***P < 0.001). Scale bars are 200 μm (**a**) and 40 μm (**b**,**c**).

We focused on the altered nuclear morphology because it could be clearly detected by lamin staining. We examined the involvement of Grp78/94 downregulation in this altered morphology by injecting adeno-associated virus (AAV) vector encoding EmGFP fused with miR RNAi for Grp78 and Grp94 (EmGFP-miR-Gpr78/94) in brain stem to knock down both Grp78 and Grp94. The AAV for non-targeting miR RNAi sequence (NT-2) was used as a control. EmGFP expression was observed in trigeminal motor neurons labeled with anti-ChAT (Fig. 4a). We then confirmed effective downregulation of Grp78/94 using AAV miR vector in these neurons (Fig. 4b). Interestingly, lamin staining revealed that in contrast to the NT-2 expressing neurons containing round-shaped nuclei, the Grp78/94-knockdown neurons contained nuclei that were relatively small and elongated (Fig. 4c). Image analysis of lamin stain by CellInsight revealed that the nuclei of Grp78/94-knockdown neurons were smaller and more elongated than those of NT-2-expressing neurons at two weeks after injection, and these differences became more significant at four weeks (Fig. 4d,e). Taken together, these data indicate that knockdown of Grp78/94 induced abnormal nuclear morphology in motor neurons, and they support the involvement of Grp78/94 downregulation in motor neuron degeneration caused by NF-Y inactivation.

### Ub/p62 accumulation in striatal and cerebellar neurons upon NF-Y inactivation

**Figure 5:**
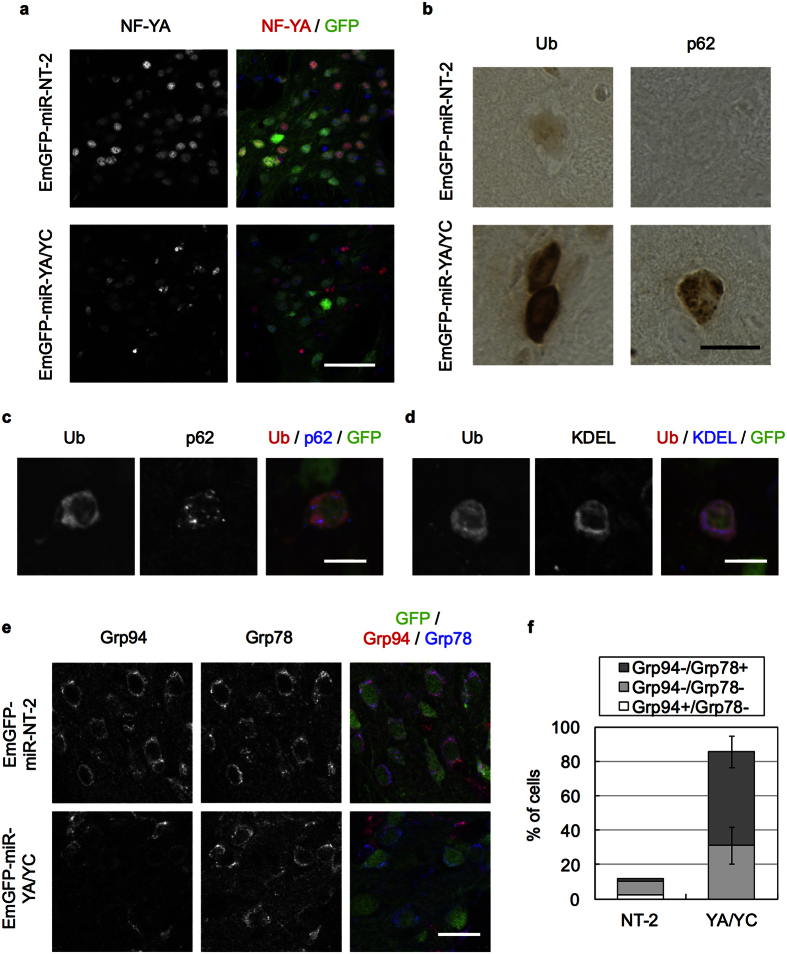
Knockdown of NF-YA and NF-YC in striatal medium spiny neurons induces Ub accumulation. AAV encoding EmGFP fused with miR-YA/YC (tandem miR RNAs against NF-YA and NF-YC) or NT-2 (non-targeting control) was injected into striatum of wild type B6 mice. Mice were fixed at 3 weeks (**a**,**e**) or 6 weeks (**b**–**d**) after injection and coronal sections were stained with antibodies as indicated. (**a**) Co-stain of the sections with NF-YA and GFP. Nuclear NF-YA signals were reduced in GFP-positive, NF-YA/NF-YC-knockdown MSNs. (**b**) Accumulation of Ub and p62 in MSNs by knockdown of NF-YA/NF-YC. (**c**,**d**) Co-staining of the sections of EmGFP-miR-YA/YC-injected brain with Ub and p62 or KDEL. Note the co-localization of Ub with KDEL but not with p62 in MSNs. (**e**) Co-staining of the sections with GFP, Grp94 and Grp78. Grp94 but not Grp78 was preferentially reduced in GFP-positive, NF-YA/NF-YC-knockdown cells. (**f**) Quantification of cells negative for Grp94 and/or Grp78 staining among the GFP-positive cells. Values are means of two (NT-2) or four (YA-YC) experiments. Note that Grp94 was lost around 90% of the NF-YA/NF-YC-knockdown cells despite loss of Grp78 in 30% of the cells. Scale bars are 50 μm (**a**), 20 μm (**b**,**e**) and 10 μm (**c**,**d**).

The lack of Ub/p62 accumulation and the loss of both ER chaperones in motor neurons suggest that NF-Y inactivation leads to neuronal type–dependent pathologies in CNS neurons. To confirm this, we further inactivated NF-Y in other types of neurons including striatal MSNs and cerebellar Purkinje cells. For this purpose, we used AAV vector encoding EmGFP fused with miR RNAi for NF-YA and NF-YC (EmGFP-miR-YA/YC)^2^ and injected it into striatum. The AAV for EmGFP-miR-NT-2 was used as a control. The infected cells were detected by GFP fluorescence in the striatum (Supplementary Fig. 4a). We confirmed reduced anti-NF-YA staining by AAV for miR-YA/YC but not by that for NT-2 in striatal MSNs (Fig. 5a), supporting gene knockdown by AAV vector. Notably, Ub accumulated diffusely whereas p62 accumulated as puncta in NF-YA/YC knockdown cells (Fig. 5b and Supplementary Fig. 4b). Confocal analysis suggested that Ub co-localized with KDEL rather than with p62 (Fig. 5c,d). Cytoplasmic accumulation of membrane proteins such as APP and CPE was also observed (Supplementary Fig. 4c,d). In addition, the NF-YA/YC knockdown cells showed preferential reduction of Grp94 (Fig. 5e). Quantification revealed ~90% of knockdown cells lost Grp94 expression, whereas around 30% of cells lost both Grp94 and Grp78 expressions (Fig. 5f). Thus, NF-YA/YC knockdown in MSNs induced Ub/p62 accumulation and selective reduction of Grp94, which closely resembles the phenomena observed in NF-YA-deletion pyramidal neurons^2^.

**Figure 6:**
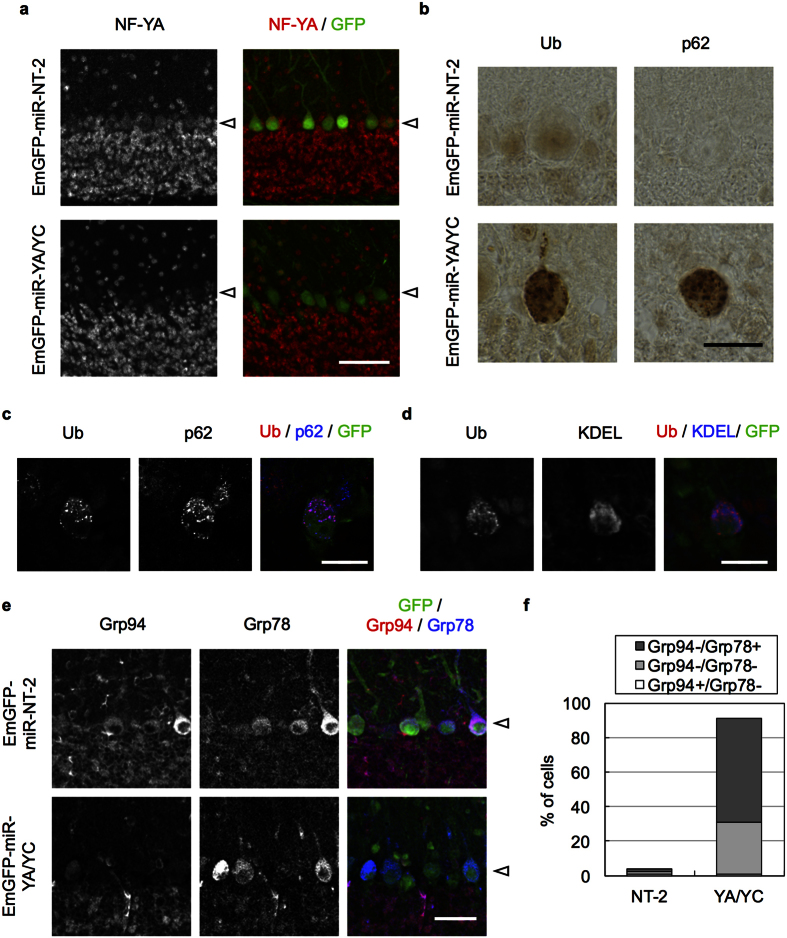
Knockdown of NF-YA and NF-YC in cerebellar Purkinje cells induces Ub accumulation. AAV encoding EmGFP fused with miR-YA/YC (tandem miR RNAs against NF-YA and NF-YC) or NT-2 (non-targeting control) was injected into cerebellum of wild type B6 mice. Mice were fixed at 2 weeks after injection and coronal sections were stained with antibodies as indicated. (**a**) Co-stain of the sections with NF-YA and GFP. Nuclear NF-YA signals were reduced in GFP-positive, NF-YA/NF-YC-knockdown Purkinje cells. (**b**) Accumulation of Ub and p62 in Purkinje cells by knockdown of NF-YA/NF-YC. (**c**,**d**) The sections of EmGFP-miR-YA/YC-injected brain were co-stained with Ub and p62 (**c**) or KDEL (**d**). Note the co-localization of Ub with p62 rather than with KDLE in Purkinje cells. (**e**) Co-staining of the sections with GFP, Grp94 and Grp78. Grp94 but not Grp78 was preferentially reduced in GFP-positive, NF-YA/NF-YC-knockdown cells. (**f**) Quantification of the cells negative for Grp94 and/or Grp78 staining among the GFP-positive cells. Values are means of two experiments. Note that Grp94 was lost around 90% of the NF-YA/NF-YC-knockdown cells despite loss of Grp78 in 30% of the cells. The Purkinje cells are indicated by arrowheads. Scale bars are 50 μm (**a**), 20 μm (**b**,**e**) and 10 μm (**c**,**d**).

To obtain further insight, we next injected the AAV vectors in cerebellum. Relatively high EmGFP expression was observed in Purkinje cells (Supplementary Fig. 5a) and NF-YA signal was lost in the cells expressing EmGFP-miR-YA/YC (Fig. 6a). We again observed accumulation of Ub and p62 (Fig. 6b and Supplementary Fig. 5b), although the Ub staining was not diffuse as in striatal neurons; the Ub puncta clearly co-localized with p62 rather than with KDEL (Fig. 6c,d). APP and CPE also accumulated in these Purkinje cells (Supplementary Fig. 5c,d). We then observed preferential downregulation of Grp94 in NF-YA/YC knockdown Purkinje cells (Fig. 6e,f). Collectively, these observations indicate that, as seen in cerebral pyramidal neurons, NF-Y inactivation induces Ub/p62 pathology with selective Grp94 downregulation in MSNs and Purkinje cells.

### Additional downregulation of Grp78 suppressed Ub accumulation induced by NF-Y inactivation in striatal neurons

**Figure 7:**
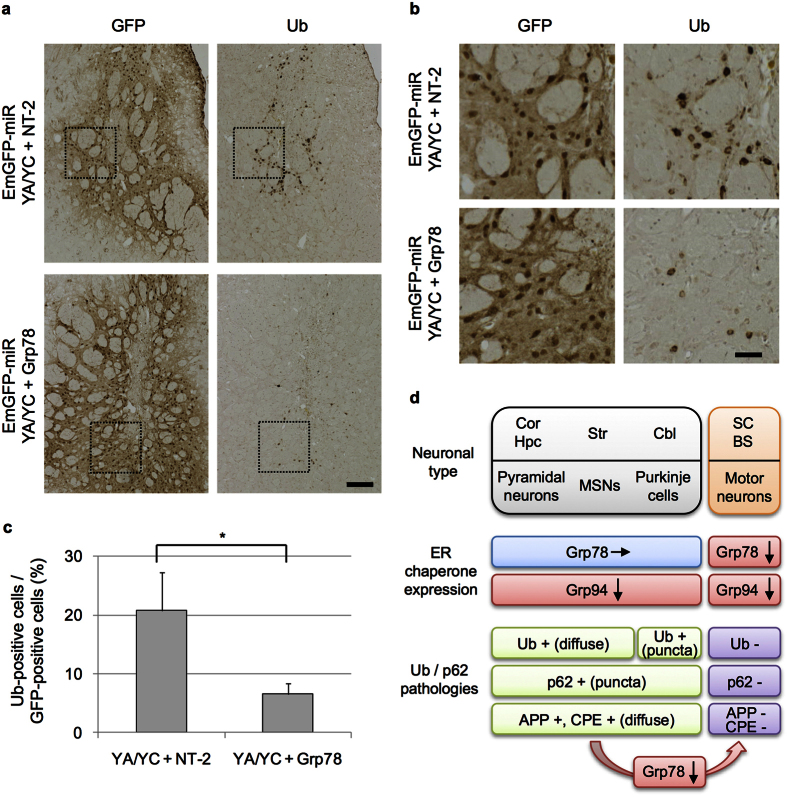
Suppression of Ub accumulation by additional knockdown of Grp78 in NF-YA/NF-YC knockdown striatal neurons. (**a**) AAV-EmGFP-miR-YA/YC was mixed with AAV-EmGFP-miR-NT-2 or -Grp78 at the ratio of 1:1 and was bilaterally injected into striata of wild type B6 mice. Mice were fixed at 3 weeks after injection and processed for cryosectioning in the coronal plane. Sequential sections were used for staining with anti-GFP and Ub antibodies. (**b**) Enlarged images of boxed regions in (a). Note the suppression of Ub accumulation induced by NF-YA/NF-YC knockdown by additional knockdown Grp78. (**c**) Number of cells positive for GFP or Ub on the sections were counted and ratios of Ub-positive cells to GFP-positive cells were calculated. More than 1900 of GFP-positive cells were counted for each mouse. Values are means + s.d. of three mice data (*P < 0.05, t-test). (**d**) Summary of the pathological responses upon NF-Y inactivation in CNS neurons. Our previous data of NF-YA deletion in pyramidal neurons of cortex (Cor) and hippocampus (Hpc) were included^2^. Similar to the pyramidal neurons, MSNs in striatum (Str) and Purkinje cells in cerebellum (Cbl) showed selective downregulation of Grp94 and accumulation of Ub, p62 and membrane proteins including APP and CPE. A slight difference was observed for the Ub accumulation pattern among these neurons. In contrast, the motor neurons in spinal cord (SC) and brain stem (BS) showed downregulation of both Grp78/94 ER chaperones and no accumulation of Ub, p62 or membrane proteins. The Ub accumulation in MSNs induced by NF-Y inactivation was suppressed by additional downregulation of Grp78.

Induction of Ub/p62 accumulation but slight downregulation of Grp78 in MSNs and Purkinje cells after NF-YA/YC knockdown led us hypothesize that difference in Grp78 expression determines the pathological phenotype caused by NF-Y inactivation. If so, additional downregulation of Grp78 in these neurons after NF-Y inactivation could suppress Ub/p62 pathology. To clarify this, AAV vector for miR-Grp78 or -NT-2 was co-injected with the vector for miR-YA/YC in striata of wild type mice. Compared with the miR-NT-2-co-injected controls that induced distinct Ub accumulation, co-injection of miR-Grp78 suppressed Ub accumulation in striatal neurons (Fig. 7a,b). Quantification revealed clear reduction of Ub-positive cells by additional Grp78 knockdown compared with the controls (Fig. 7c). These data indicate that Grp78 downregulation suppressed Ub pathology caused by NF-Y inactivation in striatal neurons, and imply that Grp78 is one of the critical factor mediating differential pathologies in CNS neurons after NF-Y inactivation (Fig. 7d).

## Discussion

Although previous studies clarified significance of NF-Y in neuronal maintenance, degeneration and differentiation^2^,^6^,^19^,^20^,^21^, whether the ubiquitous NF-Y has a conserved role in multiple CNS neurons remains unclear. By region-specific inactivation of NF-Y in adult mice CNS, we here observed neuronal type-specific neuropathologies in different CNS neurons (Fig. 7d); the NF-Y inactivation in striatal MSNs and cerebellar Purkinje cells led to cytoplasmic accumulation of Ub and p62, but not in somatomotor neurons. Subsequent analysis focusing on ER chaperones revealed that MSNs and Purkinje cells lost only Grp94 whereas motor neurons lost both Grp78 and Grp94. We further showed that knockdown of both Grp78 and Grp94 in motor neurons recapitulated the nuclear pathology observed by NF-YA knockout, suggesting involvement of their downregulation in the motor neuron degeneration. Importantly, in striatal MSNs additional downregulation of Grp78 suppressed Ub pathology induced by NF-Y inactivation, implying a critical role of Grp78 in mediating different neuropathologies in the CNS. These data indicate that NF-Y has cell type-specific roles in protein homeostasis and neuronal maintenance by regulating different sets of ER chaperones in the CNS (Fig. 7d).

So why did motor neurons show distinct neuropathologies compared with other types of neurons? Considering the central roles of Grp78 in ER function and the stress response^29^,^30^,^31^, we speculate that additional loss of Grp78 in motor neurons would be deleterious for cells and may induce a kind of acute neurodegeneration without abnormal protein accumulation. Regarding the selective regulation of Grp78 expression, a difference in NF-Y binding may not be the reason because NF-Y binding to the Grp78 promoter was broadly observed, even in the mouse cortex^2^ where NF-Y inactivation did not induce distinct Grp78 downregulation. Interestingly, by checking *in situ* hybridization data of Allen Brain Atlas (http://www.brain-map.org/)^32^, we noticed relative high expression of mRNAs for Grp78 (Hspa5: 69735138) and Grp94 (Hsp90b1: 79393537) in motor neurons of facial and trigeminal nuclei in brain stem. In addition, our immunohistochemical analysis indicates relatively high levels of Grp78/94 proteins in brain stem motor neurons compared with the neurons in cortex, striatum and cerebellum (data not shown). These data suggest that motor neurons show higher expression of Grp78/94 among the CNS neurons we examined. Thus, the underlying mechanism for expression of these ER chaperones may be different in motor neurons and NF-Y might be actively involved in this, which may lead to differential downregulation of these proteins after NF-Y inactivation in the CNS.

Notably, Grp78 downregulation upon NF-Y inactivation is also reported in several cultured cell lines^27^,^28^, suggesting dependency of Grp78 expression on NF-Y is different among the cell types and other transcription factors may contribute Grp78 expression in the neurons other than motor neurons. Indeed, HSF1, a critical regulator for stress-induced expression of cytoplasmic chaperones such as HSP70 has been reported to be differentially expressed in CNS neurons^33^. Precise analysis focusing on other transcription factors may lead to identification of the mechanism underlying differential regulation of Grp78 expression. We further found that NF-YA knockout in motor neurons induced loss of PDI, an enzyme catalyzing disulfide bonds to mediate protein folding in ER, implying that PDI expression is also regulated by NF-Y in motor neurons. This notion is supported by the observations in other contexts that NF-Y binds to the PDI promoter in cultured cell line^26^ and NF-YA knockout downregulates PDI in mouse liver^34^. In contrast, the unaffected expression of the ER membrane protein Sec61β suggests no global impairment of ER protein expression due to NF-YA deletion. Collectively, these observations suggest that NF-Y is a crucial regulator for expression of multiple ER chaperones in motor neurons and its dysfunction may result in severe disruption of ER homeostasis, leading to neurodegeneration.

Although the differential downregulation of Grp78/94 could be a potential determinant of different neuropathologies in CNS neurons, how loss of Grp78/94 mediates motor neuronal pathologies such as abnormal nuclear morphology remains unclear. In addition, it is still uncertain how NF-Y inactivation induces Ub/p62 accumulation on ER in the neurons with intact Grp78 expression, because downregulation of Grp94 is insufficient to induce their accumulation in cerebral pyramidal neurons^2^. Interestingly, the Ub pathology is variable among the Grp78-remaining neurons; diffuse distribution in pyramidal and MSNs but punctate distribution in Purkinje cells. Further studies focusing on NF-Y-downstream targets will be necessary to clarify the differential pathologies of CNS neurons after NF-Y inactivation.

In summary, targeted disruption of NF-Y in mouse CNS neurons clarify the differential regulation of ER chaperone expression and neuronal maintenance by NF-Y. To our knowledge, this is the first report describing the cell type-specific role of ubiquitous transcription factor in the CNS. Because neuronal significance has been reported for other ubiquitous factors^3^,^4^,^5^,^6^,^7^,^8^,^9^,^10^, further studies focusing on these factors may provide additional mechanisms underlying generation/maintenance of various types of neurons in the CNS. This could also lead to identification of novel mechanisms underlying cell/tissue-specific degeneration often observed in many neurodegenerative diseases and novel therapeutic targets for these diseases.

## Methods

### Mice

The mouse experiments were approved by the animal experiment committees at RIKEN Brain Science Institute and Doshisha University. Mice were maintained and bred in accordance with guidelines of RIKEN and Doshisha University. All methods were performed in accordance with the guidelines and regulations of RIKEN and Doshisha University. The NF-YA flox mice^13^ were maintained on a C57BL6 (B6) background by mating with female B6 mice. The motor neuron–specific VAChT-cre transgenic mice (VA-cre; Fast line) harboring a transgene containing cre recombinase under the VAChT promoter^24^ were developed by Dr Misawa (Keio University) and Dr Takahashi (Kyoto University) and provided by Dr Yamanaka (Nagoya University). RNZ (ROSA26-loxP-STOP-loxP-nlsLacZ) mice^25^ were provided by Dr Itohara (RIKEN BSI). All mice were maintained on a B6 background. For generation of motor neuron–specific NF-YA knockout mice, NF-YA flox/+; VA-cre; RNZ mice were first generated and crossed with NF-YA flox/flox mice. The sequences of primers used for genotyping were described previously^2^. The NF-YA knockout pups were obtained mostly at the expected Mendelian ratios. We used only male mice for the experiments. The age of the mice used for analyses is described in the figure legends. For the tremor test, the mice were placed in a suspended plastic box with an accelerometer (MVP-RF8-HC, MicroStone) at the bottom. The motion of the mice was recorded for 3 min at a sampling rate of 1 kHz, and the recorded data were transformed to amplitude at frequencies (0–100 Hz) by fast Fourier transform, as previously described^35^,^36^.

### Antibodies

Primary antibodies for Grp78 (610978) and CPE (610758) were from BD (Transduction); GFAP (Z0334) and Ub (Z0458) from DAKO; Grp94 (SPA-850), PDI (SPA-891) and Hsp40 (Hdj1) (SPA-400) from Enzo; KDEL (PM059) and p62 (PM045) from MBL; APP (MAB348), ChAT (AB144P), Sec61β (07-205) and Ub (MAB1510) from MILLIPORE; HSP 60 (K-19) (sc-1722), NF-YA (sc-10779), Hsp90α (sc-8262) and Hsp70 (W27) (sc-24) from Santa Cruz; GFP (ab13970) and LacZ (ab9361) from abcam; Iba1 (019-19741) from WAKO; LacZ (200-4136) from Rockland; p62 (GP62-C) from PROGEN; Hsp40 (Hdj2) (MS-255-P) form Neomarkers; Lamin A/C (2032) from Cell Signaling.

### Histological analysis

Mice were deeply anesthetized by peritoneal injection of tribromoethanol (Avertin), and then perfused with 4% paraformaldehyde (PFA)/phosphate-buffered saline (PBS). Isolated spines and brains were further fixed overnight with 4% PFA/PBS. The spinal cord was taken out at this point. After cryoprotection with 20% sucrose/PBS and freezing in tissue mount, the tissues were processed for cryosectioning (10–20 μm). For hematoxylin staining, the tissues were stained with Mayer’s Hematoxylin for 30–60 sec. Immunohistochemistry and immunofluorescence analysis were performed as described previously^8^,^37^. Briefly, the sections were autoclaved in 10 mM citrate buffer (pH 6.0) at 120 °C for 5 min, treated with 0.01% H_2_O_2_/methanol at room temperature for 30 min and blocked with 5% skim milk/TBST (20 mM Tris–HCl, pH 8.0, 150 mM NaCl, 0.05% Tween20) for 1 hr. The sections were then incubated with a primary antibody diluted with TBST containing 0.1% bovine serum albumin (BSA) overnight at 4 °C. For immunohistochemistry, they were then incubated with a secondary antibody conjugated with horseradish peroxidase (Vector), and then with ABC reagent (Vector), followed by detection with diaminobenzidine (DAB). For immunofluorescence, the sections were incubated with a secondary antibody conjugated with Alexa Fluor dyes (Molecular Probes). Images were obtained on a CCD camera-equipped Olympus microscope (AX80), Keyence microscope (BZ-9000 and BZ-X710), Olympus (FV1000) and Leica confocal system (TCS SP2 and SP5).

### AAV vectors and stereotaxic injection

Tandem miR RNA expression vectors for YA/YC (NF-YA and NF-YC), Grp94 (Grp94-1 and -5), Grp78 (Grp78-2 and -10) and a non-targeting control (NT-2) were generated using the pcDNA6.2-GW/EmGFP-miR vector (Invitrogen). In this system, miR RNA expression could be monitored by EmGFP expression. The oligonucleotide sequences used for construction were described previously^2^. The DNA regions encoding EmGFP-miR RNA were inserted into adeno-associated virus vector (AAV1/2-CAG-WPRE-BGH-polyA) by GeneDetect. To inject AAVs into mouse brain^38^, 6-week-old wild-type male B6 mice were first anesthetized by peritoneal injection of pentobarbital and placed in a stereotaxic apparatus (Narishige). A total of 2.7–3.0 × 10^9^ genomic particles of AAVs were injected through burr holes in the skull using a motorized microinjector equipped on the apparatus at the speed of 0.5 μl/min. For injection into striatum, the syringe needle was placed at 0.3 mm anterior to the bregma, 2 mm lateral to the sagittal suture and 2.5 mm below the skull surface. For injection into cerebellum, the needle was placed at 1.2–1.5 mm posterior to the lambda, 2 mm lateral to the sagittal suture and 1 mm below the skull surface. For injection into brain stem, the needle was placed at 1.1 mm posterior to the lambda, 1 mm lateral to the sagittal suture and 4 mm below the skull surface.

### Brain fractionation and Western Blot

Isolated spinal cords were suspended in RIPA buffer containing 20 mM Hepes at pH 7.2, 150 mM NaCl, 1 mM EDTA, 1x Complete, 1% triton X-100, 0.5% deoxycholate and 0.1% SDS with teflon homogenizer on ice. After sonication and quantification of protein concentration, the total homogenates containing 1 mg protein (T) were subjected to ultracentrifugation at 50 krpm for 30 min at 4 °C with TLA-55 rotor (Beckman). The supernatants (S1) were removed and pellets were suspended with buffer containing 500 U/ml DNaseI, 20 μg/ml RNaseA, 20 mM Tris HCl, pH 7.5, 5 mM MgCl_2_ and 1 mM CaCl_2_ and incubated at 37 °C for 30 min. After centrifugation as above and removal of supernatants (S2), the pellets were sonicated in 150 μl of 1% sarcosyl in RIPA buffer and centrifuged as above. After removal of the supernatants (S3), the pellets were further solubilized with 4% sarcosyl in RIPA buffer (S4) as above. Remaining pellets (P) were then solubilized with 100 μl of formic acid at 37 °C for 1 hr and dried by vacuum centrifugation. After boiling in SDS sample buffer, these fractions (T, S1-S4 and P) were subjected to SDS-PAGE and Western blotting as described previously^39^. Chemiluminescent signals were obtained and quantified using ImageQuant LAS-4000 (GE).

### Electron microscopy (EM)

For EM^40^, mice were perfused with 0.1 M phosphate buffer (pH 7.4) containing 2% PFA and 2.5% glutaraldehyde. Spinal cords were sectioned at 500 μm, osmicated with 1% OsO4 in phosphate buffer, dehydrated through a gradient series of ethanol, and then embedded in epoxy resin (Epon 812, TAAB). Semi-thin sections (500 nm thick) were first prepared and stained with toluidine blue. They were then processed for ultra-thin sectioning (80 nm thick) with an ultramicrotome (Ultracut UCT or UC6, Leica), and collected on 200-mesh uncoated copper grids. After counterstaining with uranyl acetate and lead citrate, the sections were examined with Hitachi HT7700 electron microscopy.

### Cell image analysis

For quantitative analysis of ChAT-positive cells in cervical spinal cords, eight cryosections (20 μm thick) were stained with anti-ChAT and the number of stained cells per section was quantified. For quantitative analysis of ER chaperone expression in motor neurons of cervical cords, cryosections from four age-matched mice were co-stained with LacZ, Grp78 and Grp94. After obtaining confocal images, we picked up all of the LacZ-positive cells and categorized them into three groups based on the staining of Grp94 or Grp78: no signal (−), background level (+/−) or similar to control level (+). Ratios to total counted cells were shown. For quantitative analysis of nuclear morphology in Grp78/94-knockdown trigeminal motor neurons, cryosections containing trigeminal nuclei were stained with antibodies for GFP, lamin A/C and ChAT, and fluorescence images were obtained by confocal microscopy. The images were then analyzed by Thermo Scientific CellInsight NXT to quantify nuclear area and elongation (ratio of long to short axis) in the GFP-positive trigeminal motor neurons. For quantitative analysis of GFP- or Ub-positive cells, the cells were first selected and quantified using ImageJ software^41^.

### Statistical analysis

For comparison between two sample groups, data were first analyzed by F-test. For P < 0.05, the data were analyzed by unpaired Student’s t-test (two-tailed); otherwise data were analyzed by Welch’s t-test (two-tailed). For multiple comparisons, the data were analyzed by one-way analysis of variance (ANOVA) using Prism software. Tukey’s test was performed for post-hoc tests. We considered the difference between comparisons to be significant when P < 0.05 for all statistical analyses. No data points were excluded in the analysis. All of the experiments were successfully repeated at least two times.

## Additional Information

**How to cite this article**: Yamanaka, T. *et al*. Differential roles of NF-Y transcription factor in ER chaperone expression and neuronal maintenance in the CNS. *Sci. Rep*. **6**, 34575; doi: 10.1038/srep34575 (2016).

## Supplementary Material

Supplementary Information
