# Supplementary material for: Differential roles of NF-Y transcription factor in ER chaperone expression and neuronal maintenance in the CNS
Source: Sci Rep. 2016 Sep 30;6:34575. doi: 10.1038/srep34575 (PMC5043352; doi:10.1038/srep34575)
Supplement: Supplementary Information [file srep34575-s1.pdf]

Differential roles of NF-Y transcription factor in ER chaperone expression and neuronal maintenance in the CNS

Tomoyuki Yamanaka, Asako Tosaki, Haruko Miyazaki, Masaru Kurosawa, Masato Koike, Yasuo Uchiyama, Sankar N. Maity, Hidemi Misawa, Ryosuke Takahashi, Tomomi Shimogori, Nobutaka Hattori and Nobuyuki Nukina

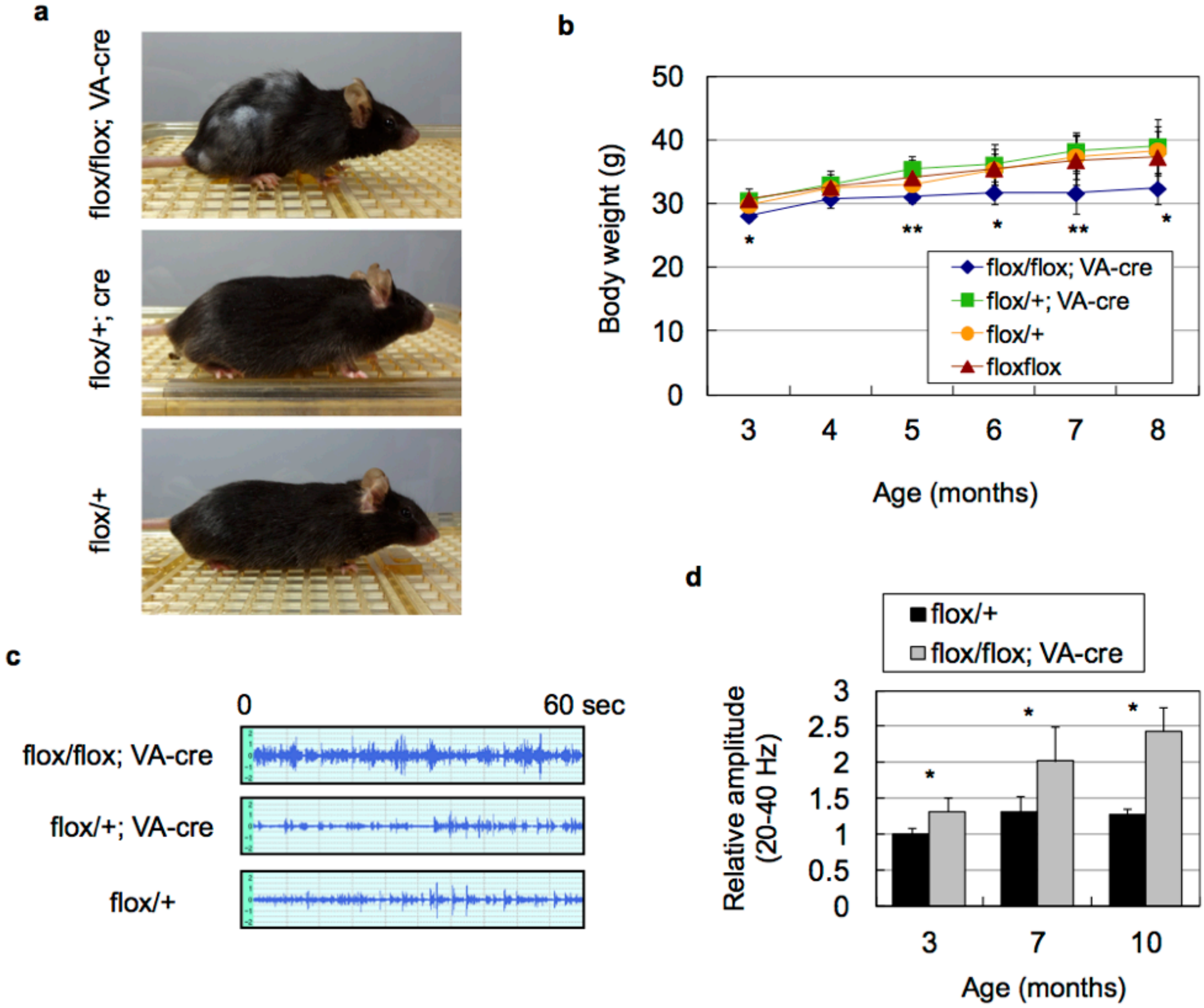

Supplementary Figure 1. Appearance of the mice with conditional deletion of NF-YA in motor neurons.

(a) Abnormal posture of NF-YA v-cko mice (flox/flox; VA-cre) compared with controls (flox/+; VA-cre and flox/+). Male mice at 10 months of age are shown. (b) Body weights of mice harboring indicated genotypes. Values are means $\pm$ s.d. Data from at least five mice are shown for each point and the data were analyzed by one-way analysis of variance (ANOVA) (*$P < 0.05$, **$P < 0.01$). (c) Motions of 10-month-old male mice with indicated genotypes were measured with an accelerometer. Raw data of vertical motions for 60 sec are shown. (d) Motions of the mice harboring flox/+ (n=4~5) or flox/flox; VA-cre (n=3) at indicated ages were recorded as in c. The obtained data were subjected to fast Fourier transformation and the amplitudes in the range of 20–40 Hz were calculated. Values are means +s.d. (*$P < 0.05$, t-test).

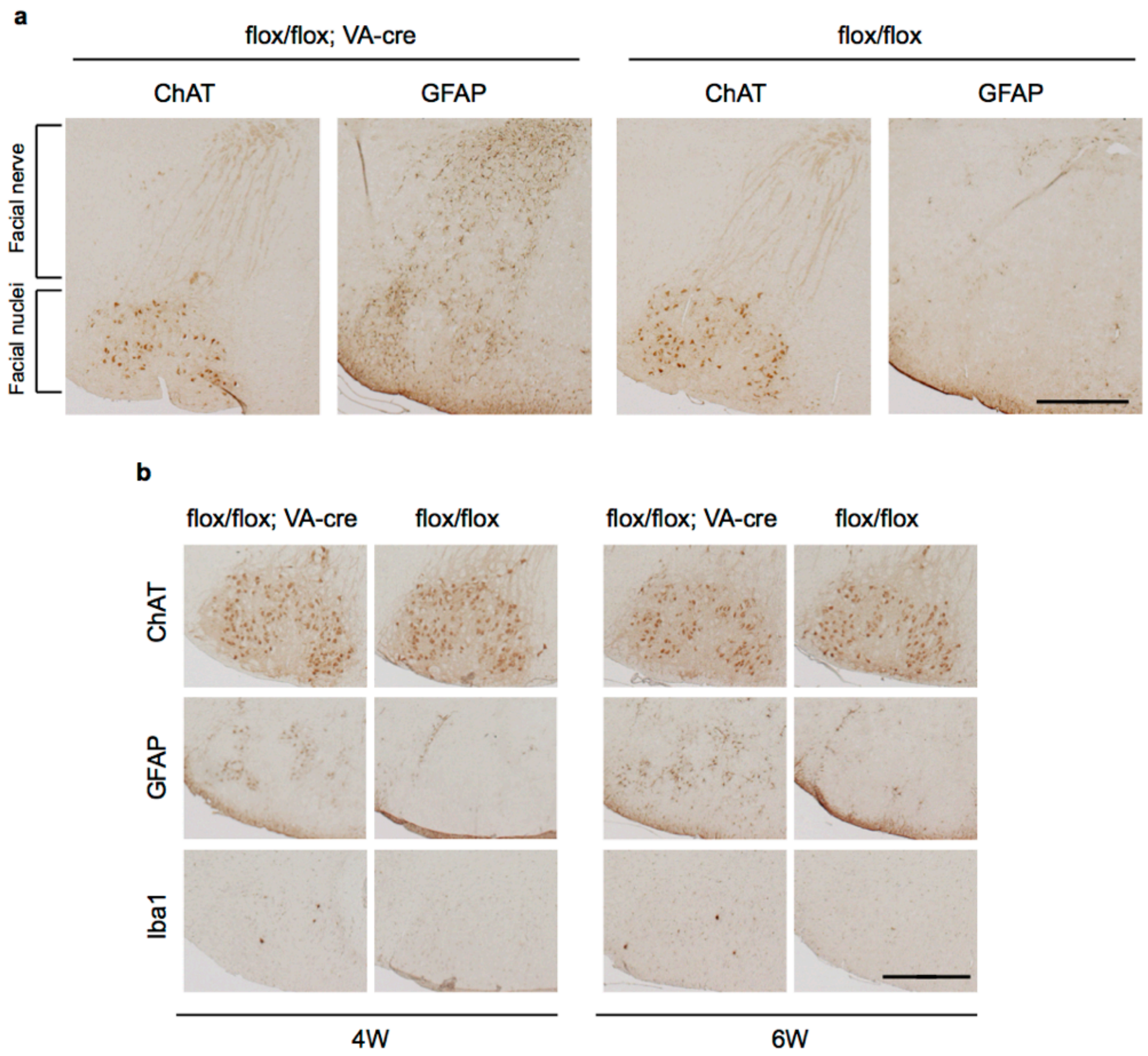

Supplementary Figure 2. Induction of gliosis in facial nuclei of NF-YA v-cko mice. Immunohistochemistry of brain stem of NF-YA v-cko (flox/flox; VA-cre) and control mice (flox/flox) mice at indicated ages. (a) Anti-ChAT and anti-GFAP staining. Note the accumulation of astrocytes in facial nuclei and its nerve of NF-YA v-cko mice at 35 weeks of age. (b) Staining of antibodies for ChAT, GFAP and Iba1 of sections from mice at 4 (left panels) and 6 (right panels) weeks of age. Note the induction of microgliosis in facial nuclei of NF-YA v-cko mice. Slight accumulation of astrocytes was also observed in these stages. Scale bars are 500 μm.

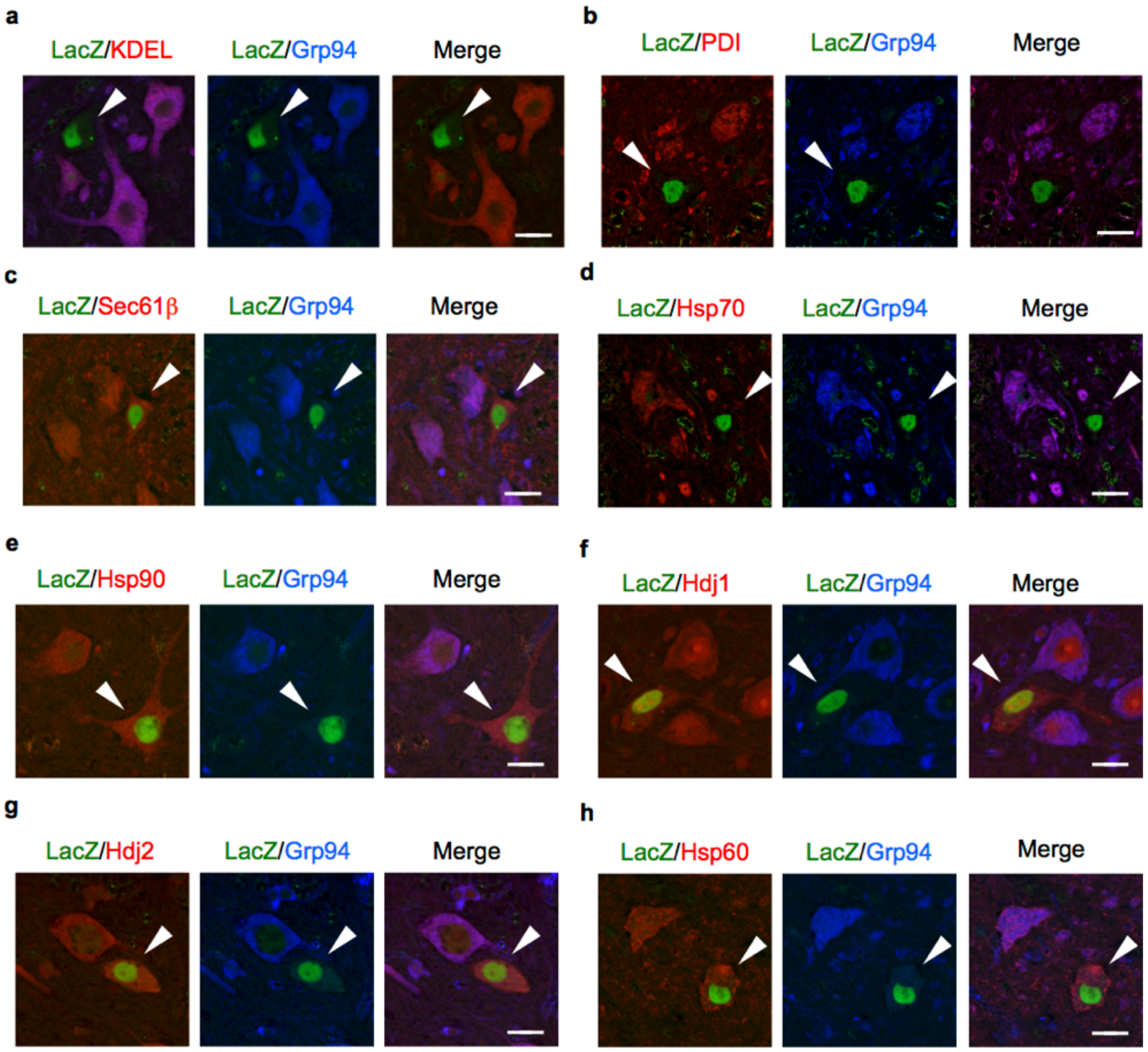

Supplementary Figure 3. Expressions of ER proteins and cytoplasmic chaperones in NF-YA v-cko motor neurons.

Cervical sections of 6-week-old NF-YA v-cko (flox/flox; VA-cre; RNZ) mice were co-stained with LacZ and Grp94 together with indicated antibodies. The NF-Y-inactive cells, characterized by nuclear LacZ stain without Grp94, are indicated by arrowheads. These cells were negative for ER luminal markers KDEL (a) and PDI (b) but not for the ER membrane marker Sec61β (c). As for cytoplasmic chaperones, the cells were positive for most of them including Hsp90 (e), Hdj1 (f), Hdj2 (g) and Hsp60 (h) except of Hsp70 (d). Scale bars are 20 μm.

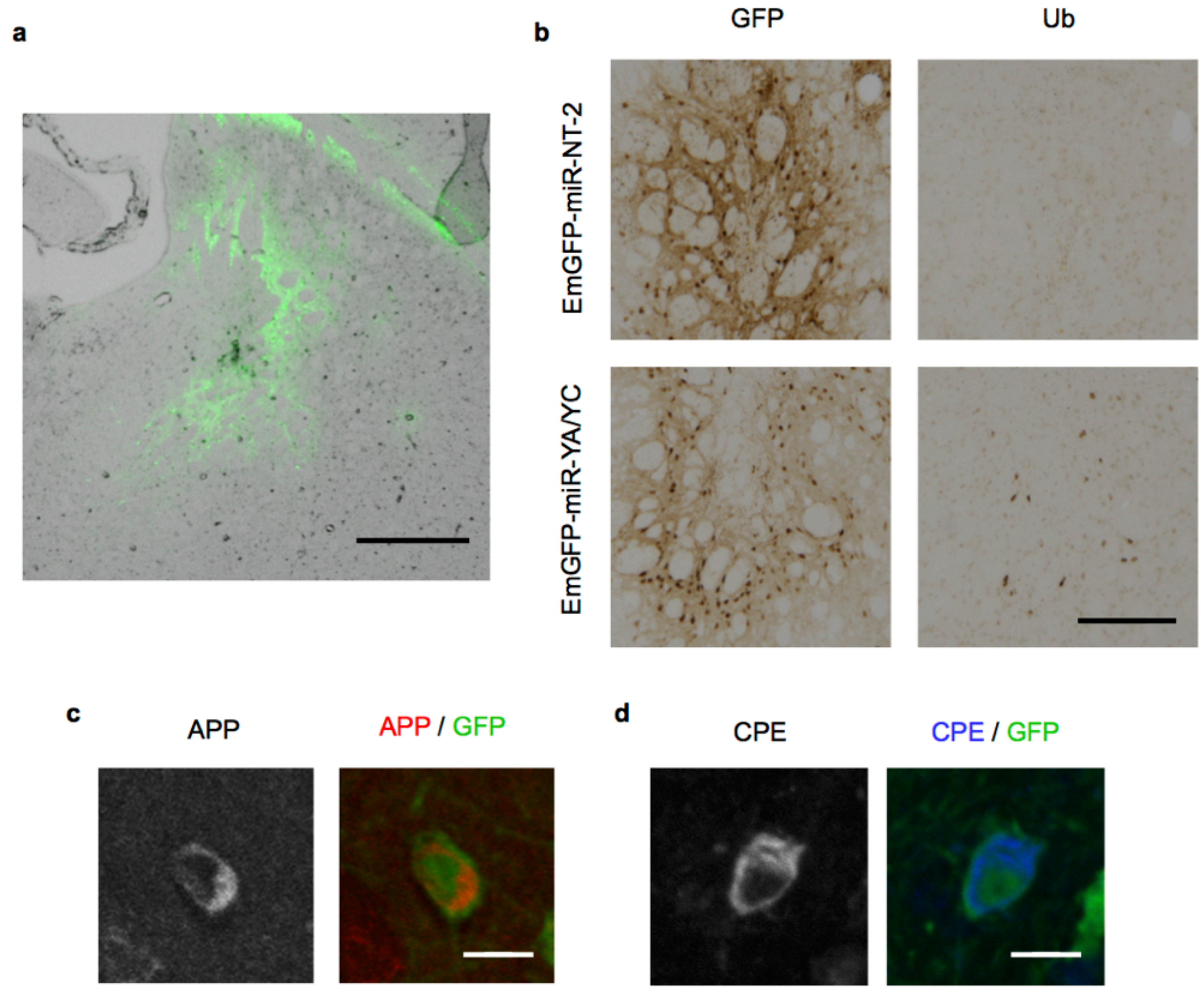

Supplementary Figure 4. Effects of AAV-mediated knockdown of NF-YA/NF-YC in striatal neurons.

AAV encoding EmGFP fused with miR-YA/YC (tandem miR RNAs against NF-YA and NF-YC) or NT-2 (non targeting control) was injected into striatum of wild type B6 mice. Mice were fixed at 3 weeks (a) or 6 weeks (b, c) after injection. (a) A merged view of GFP-fluorescence with phase contrast of coronal cryosections of AAV-EmGFP-miR-NT-2 injected brain. (b) Stain of the sections with GFP and Ub. Note the accumulation of Ub in striatal neurons by expression of EmGFP-miR-YA/YC. (c, d) Co-stain of the sections of EmGFP-miR-YA/YC-injected brain with GFP and APP (c) or CPE (d). They were accumulated in GFP-positive-striatal neurons.

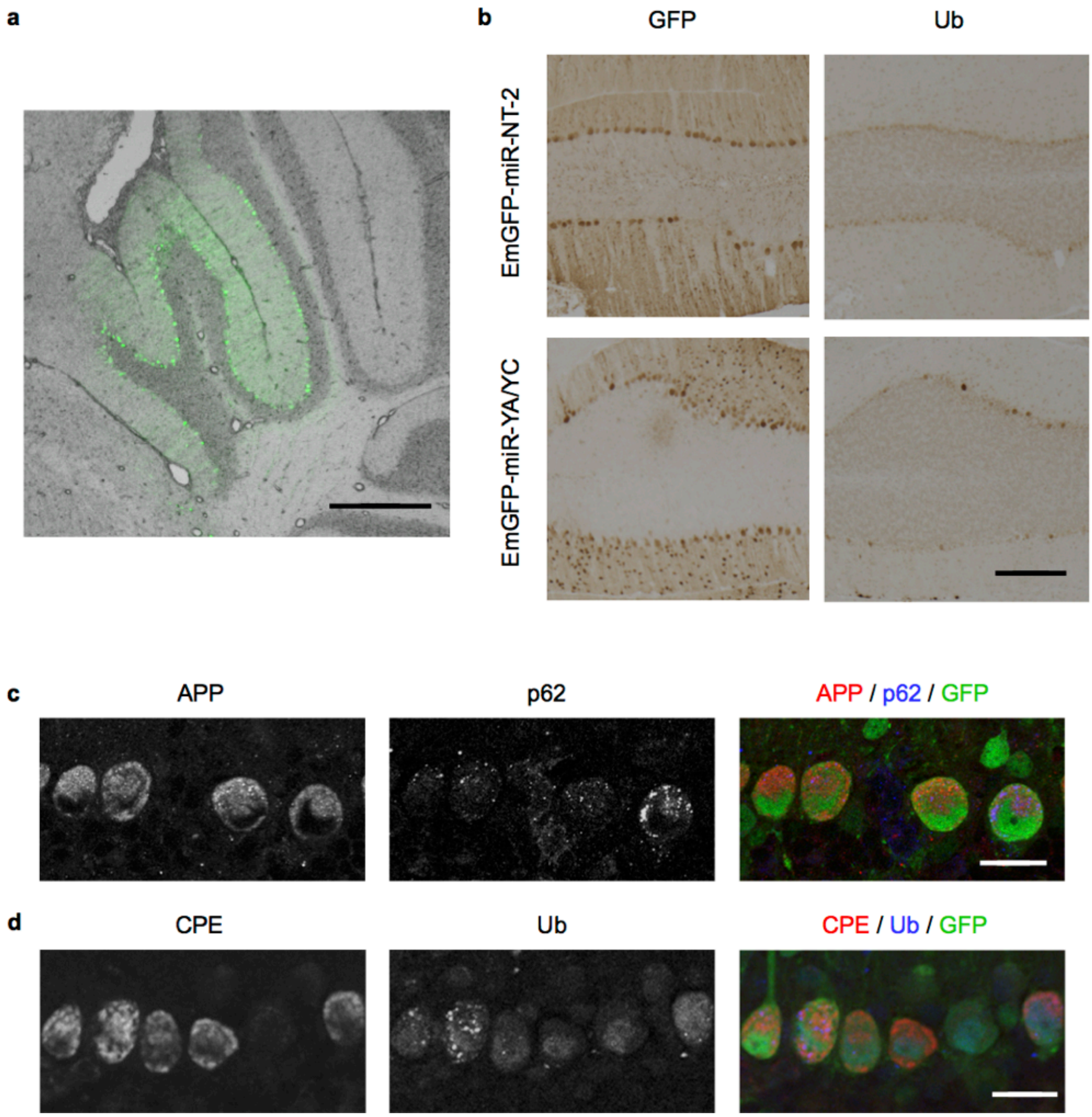

Supplementary Figure 5. Effects of AAV-mediated knockdown of NF-YA/NF-YC in cerebellar neurons.

AAV encoding EmGFP fused with miR-YA/YC (tandem miR RNAs against NF-YA and NF-YC) or NT-2 (non-targeting control) was injected into cerebellum of wild type B6 mice. Mice were fixed at 2 weeks after injection. (a) A merged view of GFP-fluorescence and phase contrast of coronal cryosections of AAV-EmGFP-miR-NT-2 injected brain. (b) Staining of the sections with GFP and Ub. Note the accumulation of Ub in Purkinje cells by expression of EmGFP-miR-YA/YC. (c, d)

The sections of EmGFP-miR-YA/YC-injected brain were co-stained with antibodies for GFP, p62 and APP (c) or those for GFP, Ub and CPE (d). Note the accumulation of APP and CPE. Scale bars are 500 μm (a), 200 μm (b) and 20 μm (c, d).
